# Bioinspired cellular membrane-derived vesicles for mRNA delivery

**DOI:** 10.7150/thno.93755

**Published:** 2024-05-19

**Authors:** Xiao Xu, Limei Xu, Jingzhi Wang, Caining Wen, Jiang Xia, Yuanmin Zhang, Yujie Liang

**Affiliations:** 1 Affiliated Hospital of Jining Medical University, Jining Medical University, Jining, China.; 2 Department of Chemistry, The Chinese University of Hong Kong, Hong Kong, China.; 3 College of Rehabilitation Medicine, Jining Medical University, Jining, China.

**Keywords:** cellular membrane-derived vesicles, exosomes, extracellular vesicle, mRNA delivery, mRNA vaccine

## Abstract

The rapid advancement of mRNA as vaccines and therapeutic agents in the biomedical field has sparked hope in the fight against untreatable diseases. Successful clinical application of mRNA therapeutics largely depends on the carriers. Recently, a new and exciting focus has emerged on natural cell-derived vesicles. These nanovesicles offer many functions, including enhanced drug delivery capabilities and immune evasion, thereby presenting a unique and promising platform for the effective and safe delivery of mRNA therapeutics. In this study, we summarize the characteristics and properties of biomimetic delivery systems for mRNA therapeutics. In particular, we discuss the unique features of cellular membrane-derived vesicles (CDVs) and the combination of synthetic nanovesicles with CDVs.

## 1. Introduction

Messenger RNA (mRNA) was initially identified in the early 1960s 1, and the synthesis of biologically active mRNA was reported in 1984 2. Functioning as a pivotal intermediary, mRNA serves to manipulate target genes, orchestrating the expression of proteins or active substances and thus assuming a critical role in the transmission of genetic information. Unlike DNA-based protein expression technology, mRNA does not need to penetrate the cell nucleus and refrains from integrating into the genome 3, 4, incurring less concern for safety. Furthermore, after spontaneous degradation, the resulting products of mRNA are efficiently recycled by the cell.

In contrast to traditional vaccines, which necessitate prolonged development time, mRNA vaccines boast a more expedited development cycle due to the simplicity of antigen substitution technology 5. The advantages of mRNA therapy were found to be particularly useful during the COVID-19 pandemic, when mRNA technology was harnessed for vaccine production, contributing to the prevention of COVID-19 6.

Despite the extensive potential of mRNA technology, one notable challenge was its inherent fragility and rapid *in vivo* degradation, which impeded its bioavailability. Consequently, developing effective delivery systems is paramount in broadening the application scenarios of mRNAs 7-11.

**Figure 1:**
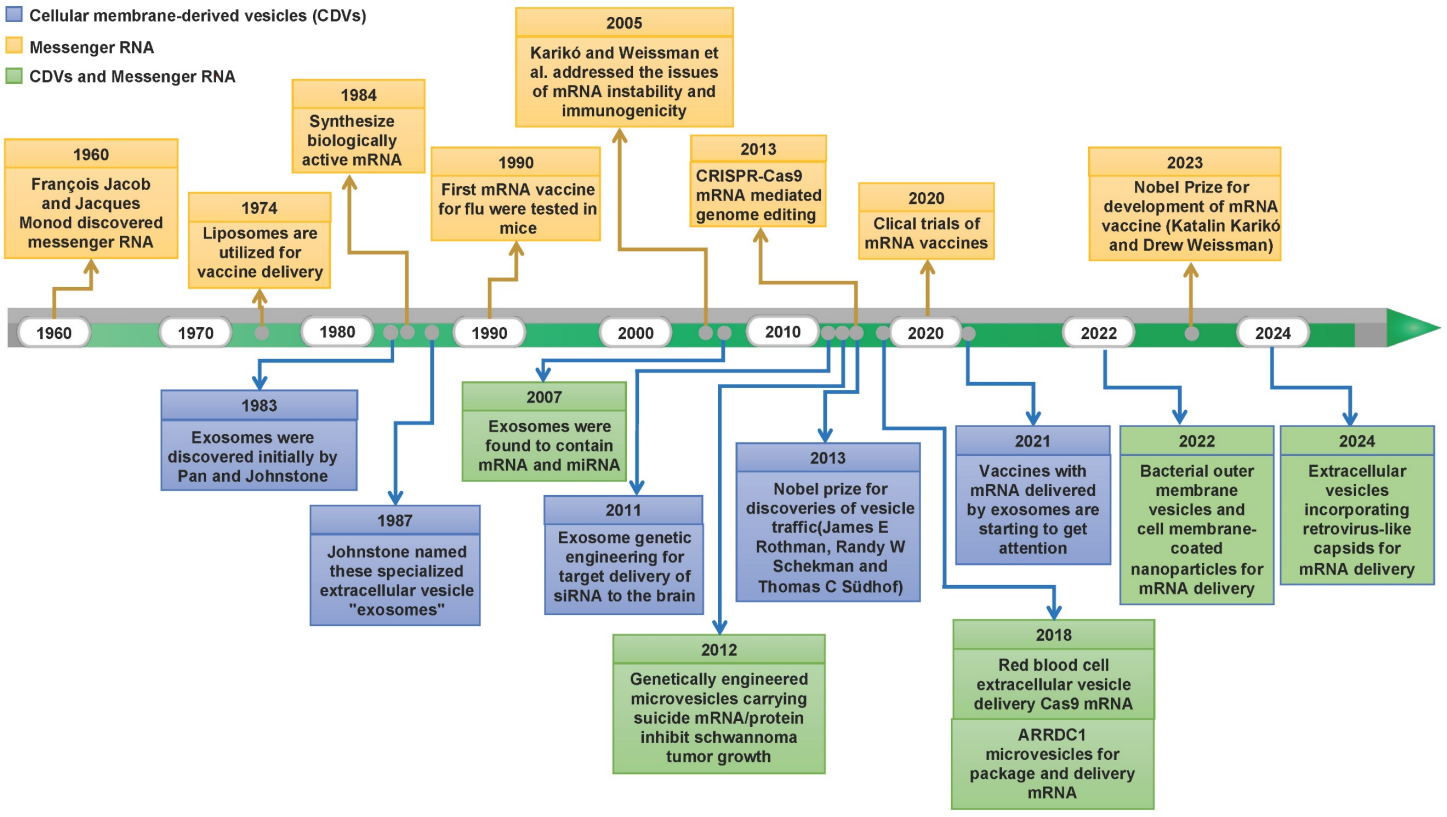
Historical timeline marking milestone discoveries in mRNA biology and CDV-based drug delivery.

Natural cellular membrane-derived vesicles (CDVs) have increasingly captivated attention owing to their endogenous origin. This affords them the advantages of eliciting low immune responses, displaying excellent cytocompatibility, and exhibiting heightened in vivo stability 12-14. Over the years, many research endeavors and experiments have flourished, propelling the burgeoning potential of mRNA technology centered on CDV-mediated discovery. **Figure 1** depicts major discoveries in RNA therapy and CDV-based drug delivery platforms. Recently, an array of cell-based nanoplatforms has been meticulously crafted, assuming a pivotal role in refining the applications of mRNA. These platforms are instrumental in augmenting the targeting precision, enabling the ingress of mRNAs into target cells, surmounting diverse physiological barriers, and thereby broadening the horizons of mRNA applications. Nevertheless, challenges persist in the delivery of mRNAs, encompassing aspects such as the methodology for constructing targeting mechanisms and the selection of nanoplatforms. Thus, in this paper, we comprehensively review the categorizations, merits, and methods of modification associated with CDVs based on their cellular origins. Furthermore, we deliberate on contemporary dilemmas and chart a course for future development, aspiring to furnish valuable insights for the innovation of mRNA technology.

## 2. Biology of CDVs

CDVs, including exosomes, microvesicles (MVs), apoptotic bodies, or outer membrane vesicles, are naturally occurring particles from cells 15, 16. CDVs exhibit dimensions, shapes, and structures similar to liposomes but possess a more intricate bilayer structure comprising a diverse array of lipids, proteins, and carbohydrates, along with internal cargoes and surface-associated molecules, often numbering in the hundreds. They assume a crucial role in facilitating short- and long-distance intercellular communication across a spectrum of pathophysiological processes. The capacity of CDVs to shuttle biomolecules to receptor cells renders them particularly appealing for drug delivery applications. Consequently, this section elucidates the mechanisms underpinning CDN biogenesis and cargo loading, concurrently exploring the physiological functions integral to these processes.

### 2.1 An overview of extracellular vesicles

**Figure 2:**
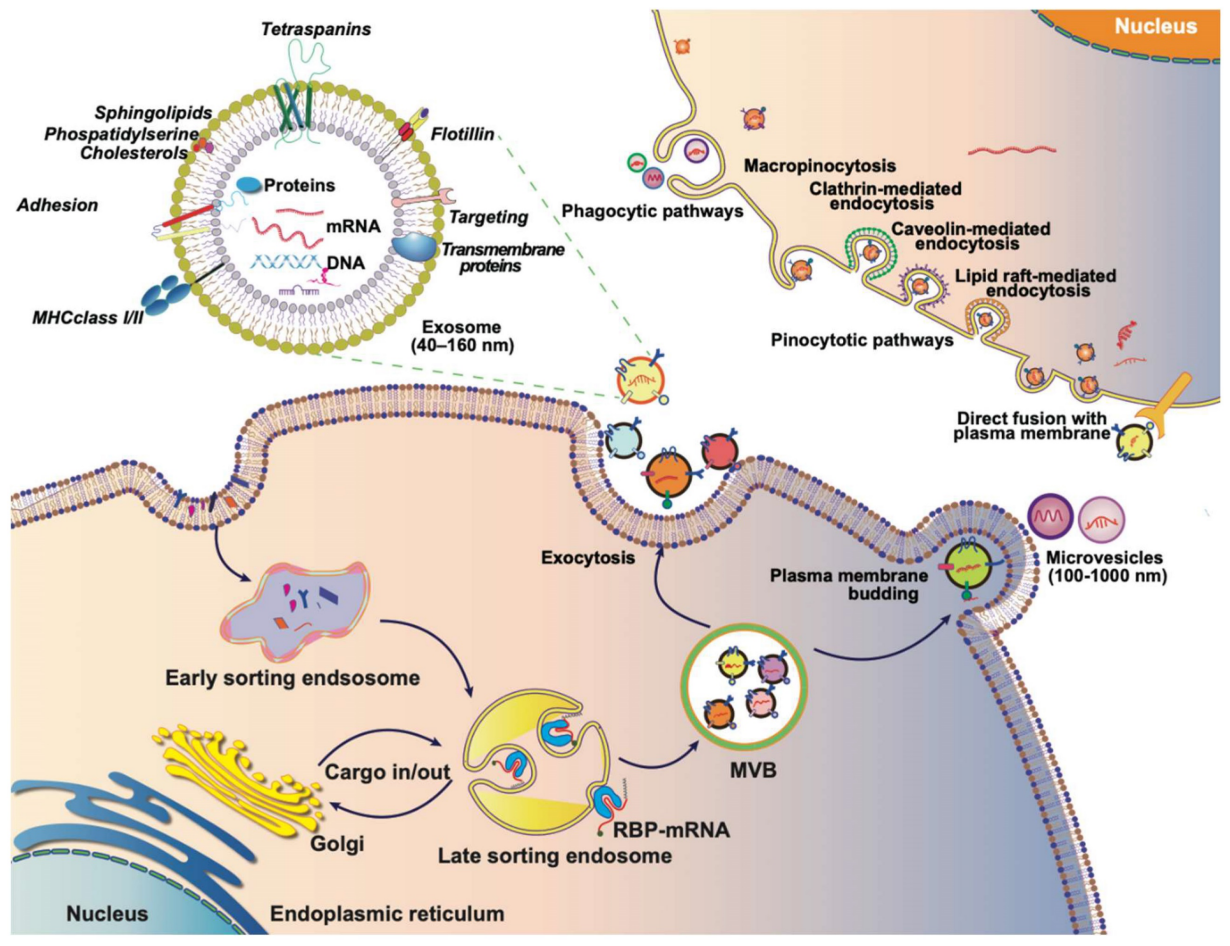
The journey of EVs: biogenesis, composition, and internalization. Exosomes typically originate from endosomes, and the endogenous cargo is selectively sorted into early endosomes, which subsequently mature into late endosomes or MVBs. As MVBs bud inwardly, they give rise to ILVs, which can then be transported to the plasma membrane. Exosomes are released into the extracellular matrix upon fusion with the plasma membrane. MVs, on the other hand, are generated through outward budding or exocytosis directly from the plasma membrane, featuring diameters ranging from 100 to 1,000 nm. Both exosomes and MVs, collectively referred to as EVs, are rich in proteins, lipids, nucleic acids, and other contents derived from the parent cell. The internalization of EVs by recipient cells can occur through various mechanisms, including phagocytosis or receptor-mediated endocytosis. Alternatively, exosomes can directly fuse with the membrane of the recipient cell, facilitating the transfer of their contents directly into the cytoplasm of the recipient cell. This multifaceted journey of exosomes, encompassing their biogenesis, composition, and modes of internalization, underscores their significance as dynamic mediators of intercellular communication.

Extracellular vesicles (EVs) are membrane-bound vesicles, including microvesicles, exosomes, apoptotic bodies, and exosome-like nanovesicles. Exosome generation commences with the formation of early endosomes through the invagination of the plasma membrane into the cell. During this process, the cargo from endocytosis concentrates in the central vesicle region of the early endosome, and the endosomal membrane undergoes further maturation. Subsequently, the endosomal membrane inwardly depresses, giving rise to intraluminal vesicles (ILVs) within multivesicular bodies (MVBs). Specific cytoplasmic components, proteins, and lipids in late endosomes are enclosed in ILVs during this phase. Following the fusion of MVBs with cell membranes, the secretion of ILVs occurs, contributing to the formation of exosomes (**Figure 2**) 17-19. The occurrence and secretion of ILVs are notably critical aspects of exosome biogenesis. The mechanism of ILV occurrence is primarily categorized into two processes: the endosomal sorting complex required for transport (ESCRT)-dependent and ESCRT-independent ontogeny. ESCRT-dependent occurrence involves sorting complexes such as ESCRT-0, ESCRT-Ⅰ, ESCRT-Ⅱ, and ESCRT-Ⅲ. The ubiquitin-binding subunit of ESCRT-0 recognizes ubiquitin-modified membrane proteins and is subsequently delivered to ESCRT-Ⅰ and ESCRT-Ⅱ. The endosome buds inwardly through interactions with ESCRT-Ⅰ, ESCRT-Ⅱ, and ESCRT-Ⅲ, forming ILVs 20. ESCRT-independent ontogeny involves the formation of ESCRT-independent MVBs, primarily facilitated by transmembrane proteins like CD63, Rab GTPases, ceramide, Flotillins, Caveolin-1, and others 21-26.

Exosome biogenesis is an intricate and relatively unexplored process, characterized by its relative inherent randomness in the composition of its inclusions. This results in the rich compositional diversity of exosomes. The heterogeneity stemming from exosome genesis does not impede their applications. Exosome composition is contingent upon the cell of origin, yet certain commonalities persist. Generally, exosome encompasses transmembrane proteins such as CD9, CD63, CD81, ESCRT proteins (Alix and TSG101), heat shock proteins (HSP70 and HSP90), lipids (cholesterol, sphingomyelin, and ceramides), cytoskeletal components (actin and myosin), as well as nucleic acids including DNA, mRNA, and non-coding RNAs 18, 20. These lipids, proteins, and nucleic acids collaboratively execute the biological functions of exosomes. Exosomes wield potent biological functions, primarily serving as carriers for cell-to-cell communication by delivering biologically active substances or facilitating signal communication. The biological activities of exosomes are intricately tied to the functions of their inclusions 24. Notably, exosomes carry mRNA, a key medium for cell-cell communication of bioactive substances or signals 19. Early studies have identified approximately 1,300 genes in mouse MC/9 and human HMC-1 exosomes. Subsequent investigations have revealed the transferability of exosomal mRNAs from mouse to human mast cell lines, emphasizing the efficacy of exosomes as mRNA carriers to other cells 27. Following this, multiple studies have identified exosomes enriched with mRNA as potential biomarkers for various diseases. For instance, the exosomal mRNA expression of EGFRvIII in tumor cells has been recognized as a diagnostic biomarker for glioblastoma 28. Additionally, serum exosomes have been found to contain an elevated presence of heterogeneous nuclear ribonucleoprotein H1 (hnRNPH1) mRNA, particularly in patients diagnosed with hepatocellular carcinoma (HCC) 29.

Furthermore, exosomes loaded with mRNAs can be delivered to cells in a targeted fashion, showing the potential as ideal vectors for gene therapy. The double-membrane structure of exosomes protects loaded mRNA from degradation by RNA enzymes in body fluids. For example, the literature showed that urinary exosomal mRNA remained stable at 4 ℃ for up to 2 weeks 30. Therefore, an in-depth exploration of the fundamental composition of exosomes holds promise for advancing our understanding of disease processes.

### 2.2 Generation of MVs and their characteristics

MVs originate directly from the plasma membrane and typically range in size from 50 nanometers to one micron in diameter 31. Initially identified as "platelet dust" or extra-nuclear granular bodies, MVs were later discovered in stimulated neutrophils, demonstrating outward budding from the plasma membrane 32. Recent studies have reported a similar process resulting in the formation of oncosomes with potentially larger diameters (up to 10 microns) in various tumor cells. Subsequently, MVs have been identified in normal cell types such as erythrocytes, leukocytes, endothelial cells, and even in urine samples 33.

The formation of MVs is contingent on the transport of sufficient amounts of phospholipid phosphatidylserine from the inner to the outer leaflet of the plasma membrane. The contraction of MVs, essential for their shedding, is facilitated by the cytoskeleton, particularly triggered by ADP-ribosylation factor 6 (ARF6) 34. Protein 1 (ARRDC1), situated in the arrestin structural domain on the cytoplasmic side of the plasma membrane, recruits the ESCRT-I complex protein TSG101, a crucial factor for MV budding and release 35. Additionally, the asymmetric distribution of lipid molecules, such as phosphatidylethanolamine, phosphatidylcholine, and phosphatidylserine across the plasma membrane, plays a pivotal role in regulating MV stabilization and exocytosis 36.

The cargo content of MVs varies based on the cell's origin, pathological and physiological state, and triggering factors 31, 37. Given that MVs are directly formed through plasma membrane detachment, cellular membranes and membrane proteins such as HSPs, integrins, cytoskeletal proteins, and tetraspanins from the mother cell are present in MVs. Annexin A1, a member of the Ca^2+^-dependent phospholipid-binding membrane protein family, has been identified as a specific marker for MVs 38. While MVs are produced by the direct shedding of the plasma membrane, the loading of contents into the vesicles is not a random process. Specific protein components are selectively included or excluded from the contents of MVs or their membranes 31. The mechanism governing this process remains largely unexplored, although the ARF-6 protein has been implicated in the MV cargo sorting process.

In addition to proteins, MVs carry various types of genetic materials, including mRNA and microRNA (miRNA). These genetic materials are transferred to recipient cells, influencing the phenotype of the target cells and playing crucial roles in processes such as coagulation, inflammation, and tumor progression 39.

### 2.3 Exosome mimics

While natural cells secrete a limited number of exosomes or MVs with inherent heterogeneity 36, the pursuit of overcoming this bottleneck has led to burgeoning research focusing on exosome-mimics. These mimics possess a lipid bilayer structure akin to cell membranes, and their nanoscale size renders them promising for diverse applications. Different synthetic approaches for these mimics are classified into top-down, bottom-up, and biohybrid technologies. Top-down strategies involve the creation of artificial exosomes starting from the cellular level. The main methods include extrusion, microfluidics, ultrasound, chemical induction, and nitrogen cavitation 40-43. While top-down strategies can yield a substantial number of exosome analogs, enhancing the overall yield, the production process tends to be more time-consuming. Bottom-up strategies involve constructing nanovesicles and gradually endowing them with specific functions. Techniques in this category include membrane protein-modified liposomes, antibody-modified liposomes, and peptide-modified liposomes 44-46. The synthetic processes, however, differ significantly from natural exosomes, limiting their ability to fully replicate the biological functions of natural counterparts. Biohybrid technologies involve the fusion of natural exosomes with artificial nanovesicles, employing methods such as co-incubation, freeze-thawing, and co-extrusion 47-49. The emergence of exosome-mimics has significantly expanded the application scenarios of cell-free therapy. This evolving field holds great promise for further research, presenting opportunities to overcome limitations and harness the full potential of exosome-based therapeutic interventions.

### 2.4 Apoptotic bodies

During apoptosis, a series of molecular events culminate in DNA breaks and cell membrane rupture, while small membrane particles called apoptotic bodies (ApoBDs) are released from the cell membrane. Apoptotic bodies mainly carry large chromosomal DNA fragments and various cytoplasmic proteins, with the function as intercellular communication. Deep sequencing of total RNA from endothelial cells derived apoptotic bodies revealed that ApoBDs has a unique protein-coding RNA profile, in which PCSK5 is the most abundant mRNA. After cellular internalization of ApoBDs, PCSK5 mRNA was transferred into the target cell 50. However, apoptotic vesicles are rarely considered for drug delivery because of their heterogeneous size distribution and complex composition. Recently, researchers isolated small apoptotic bodies (sABs) and revealed their potential advantages as a delivery system targeting the brain 51. As these sABs are more homogeneous and have fewer DNA fragments, they may revolutionize mRNA delivery for the treatment of many brain diseases.

### 2.5 Membrane-coated nanoparticles

Cell membrane-coated nanoparticles (CNPs) are a new class of nanomaterials that utilize cell membranes to encapsulate synthetic nanoparticles. This combination of the functional properties and surface markers of synthetic materials and natural cell membranes allows CNPs to mimic natural cellular interactions and offers great promise for mRNA delivery 52. A variety of cells, including platelets, erythrocytes, and cancer cells, were used to isolate cell membranes for nanoparticle encapsulation 53-57. Red blood cell membranes have been used to extend the circulation time of nanoparticles, while cancer cell and platelet membranes have been used for targeted drug delivery. Given the central role of dendritic cells (DC) in controlling the immune response, DC membrane-encapsulated nanoparticles can be used to promote the expression of mRNAs in the lymph nodes and spleen, inducing the induction of humoral and cellular immune responses 58.

CNPs can also be modified or loaded with other components to enhance biological functions. Recently, genetic engineering methods have been used to generate cell membranes with specific surface markers, thus enabling researchers to manipulate the function of cell membrane-encapsulated nano-formulations. These engineered nanoparticles can be equipped with complex surface proteins that cannot be done using traditional synthesis methods. In one study, cell membranes were genetically engineered to express a SpyCatcher membrane anchor that could covalently bind to any moiety modified with SpyTag, allowing for enhanced functionality of cell membrane-encapsulated nanoparticles 59. In another study, genetically engineered modified T-cell membranes were combined with aggregation-induced-emission nanoparticles to develop a novel photothermal therapy, which effectively avoided glioblastoma recurrence 60. Utilizing cell membranes expressing influenza A virus HA subtype H7 fusion proteins to coat mRNA-loaded nanoparticles could enable HA-mRNA-NP formulations to mimic the viral endosomal escape effect, thereby significantly increasing the levels of encoded proteins in both topical and systemic administration 58. Overall, cell membrane coating is an emerging top-down approach to endow nanocarriers with enhanced biointerfacial properties.

### 2.6 Outer membrane vesicles

Outer membrane vesicles (OMVs) are nano-sized natural vesicles formed by the outward budding of the outer membrane of Gram-negative bacteria, with a size of 30-250 nm 61. OMVs carry a large number of bacterial-derived materials, including lipopolysaccharides, peptidoglycan, membrane, cytoplasmic and cytoplasmic proteins, toxins, and nucleic acids, thereby affecting a wide range of biological processes, including virulence, horizontal gene transfer, phage infection, transport of cellular metabolites, and bacterial-bacterial or bacterial-host interactions. Currently, OMVs have been widely used as an adjuvant in vaccine production 62. In addition, it has been shown that OMV membranes are mainly composed of lipids and proteins, with a structure similar to that of cell membranes, and exhibit a very high degree of biocompatibility, which has great potential as a new type of drug delivery carrier. Presentation of homologous or heterologous antigens from different pathogens or other sources by fusion expression with scaffolding proteins on OMVs can strongly stimulate the innate immune system and promote antigen presentation and T cell activation 63. For example, *E. coli* derived OMVs that express antigens of influenza virus, Plasmodium falciparum, pneumococcus, hepatitis C virus, hepatitis B virus, etc., can stimulate the body to produce specific antibodies against these pathogenic microorganisms 64. Therefore, OMVs are ideal nanocarriers in developing mRNA vaccines against pathogenic microorganisms or cancer.

## 3. Advantages of CDV-based delivery systems

**Figure 3:**
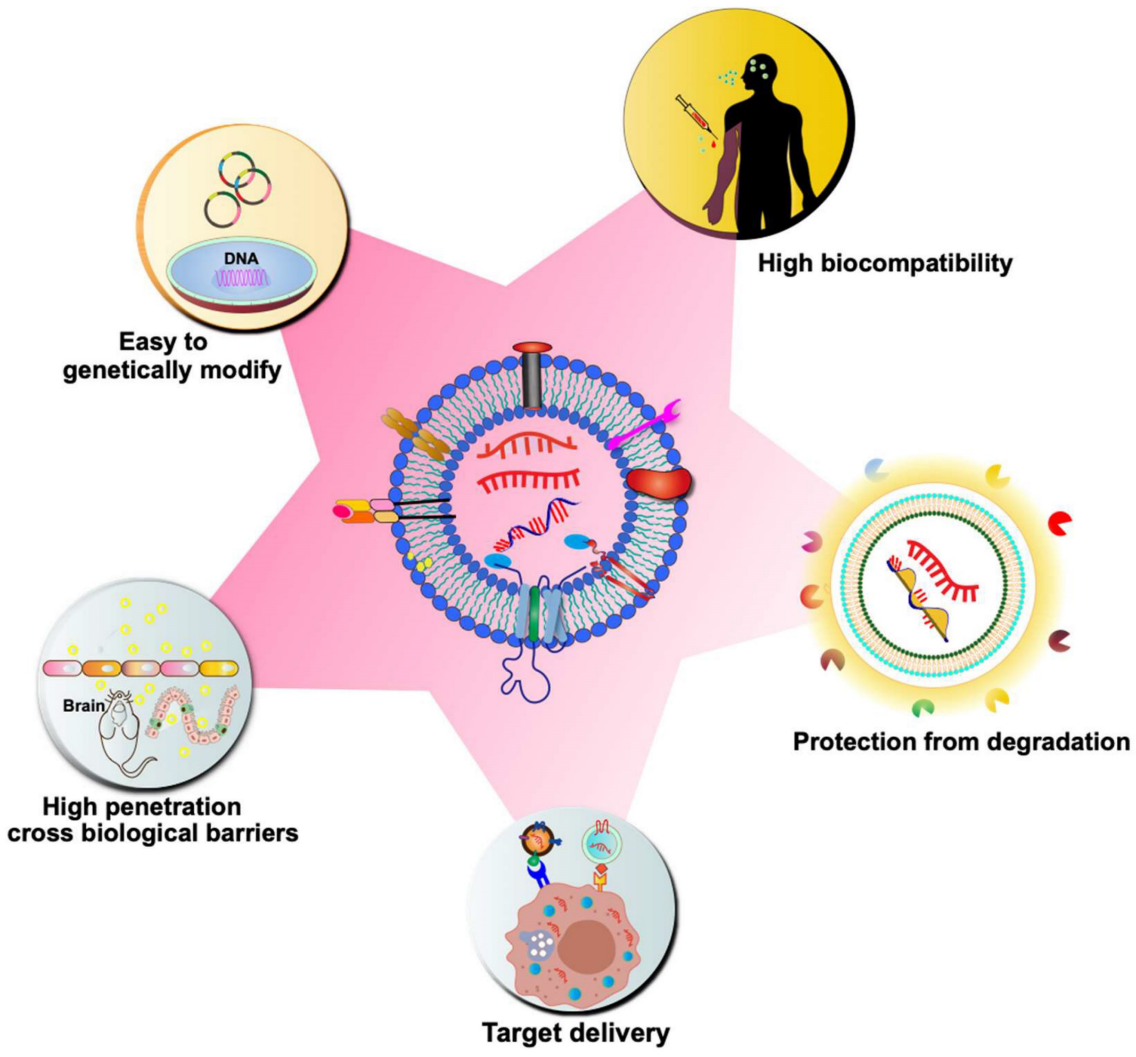
Advantages of CDVs as nano-scale drug carriers for mRNA delivery. As biological nanoparticles naturally released by cells, CDVs exhibit high biocompatibility and low immunogenicity. The lipid bilayer membrane structure of CDVs provides a protective encapsulation for nucleic acid drugs, preventing their degradation by enzymes and enhancing their stability. CDVs can be genetically modified to enhance delivery targeting and enable selective loading of mRNA. Moreover, their efficient uptake by target cells and the ability to traverse biological barriers, such as the blood-brain barrier (BBB), further underscore the potential of CDVs as a drug carrier for mRNA delivery.

Although synthetic particles, such as liposomes, have been on the market for over 20 years, their use has significant drawbacks, such as high toxicity, high clearance, and immunogenicity 65. On the contrary, CDVs are naturally occurring particles, which are highly biocompatible. As previously discussed, CDVs inherently carry genetic material for delivery to target cells, playing a crucial role in regulating gene expression in various biological and pathogenic processes. Due to their natural ability to transport genetic material, CDVs have garnered significant interest in drug delivery strategies, particularly in the realm of mRNA-based gene therapy. CDVs such as exosomes are nanoscale vesicles and carry a variety of membrane proteins that can interact with target cells, mediate their membrane fusion and endocytosis, and thus have a strong cellular uptake efficiency 66. CDVs can cross tissue boundaries and diffuse into deeper tissues to overcome various biological barriers, such as the blood-brain barrier, the blood-tumor barrier, and the cartilage tissue barrier 67, 68. Natural CDVs are rapidly cleared by the kidneys, liver, spleen, and lungs, but it has been found that altering the surface molecules of CDVs avoids circulatory clearance, for example, by blocking the scavenger receptor class A family (SR-A), which reduces clearance by the liver 69. The incorporation of different antiphagocytic molecules on the surface of exosomes leads to remaining in the circulation and tissues for a longer period of time 70. In addition, CDVs can avoid the endosomal pathway and lysosomal degradation more efficiently than synthetic carriers 71. Due to the small size and slight negative charge of exosomes, it can avoid passage through the reticuloendothelial system, which in turn reduces renal clearance and prolongs the circulation time of exosomes at the treatment site 72. Another advantage of using CDVs as delivery vehicles is their hydrophilic cores, which makes them suitable for loading soluble drugs, such as nucleic acids. The bilayer membrane effectively protects the loaded cargo, thereby preventing enzymatic degradation of the delivered nucleic acid drug. Multiple signaling molecules and co-stimulatory factors can also be loaded at the same time for delivery to specific cell types. Moreover, CDVs have low immunogenicity compared to liposomes and virus-based drug delivery systems. Compared to artificial nanoparticles, CDVs have a greater targeting ability. The surface of CDVs can be modified with various molecules to achieve targeted delivery. For instance, targeting ligands (e.g., antibodies and oligonucleotides) can be immobilized on the exosome surface through covalent bonding or electrostatic interactions, facilitating targeted delivery to specific receptor cells 73. The unique properties of CDVs will speed up mRNA therapy in the near future (**Figure 3**).

## 4. Preparation of mRNA-loaded CDVs

### 4.1 Preloading methods

The strategies for loading mRNAs into CDVs can be broadly categorized into two main groups: preloading methods and post-loading methods. Preloading methods, also known as endogenous loading methods, rely on donor cells loading mRNA cargoes into CDVs during biogenesis. A common approach is overexpressing plasmids in parental cells to obtain transcribed mRNAs. Overexpressing transcribed mRNAs promotes their enrichment in CDVs. This strategy has been employed in several studies to load different mRNAs into CDVs. For instance, the mRNA of the low-density lipoprotein receptor (Ldlr) is encapsulated into exosomes by expressing it in the donor cells. Upon transfection of the plasmid, Ldlr mRNA levels in the donor cells increase more than 100-fold compared to cells transfected with the control plasmid, resulting in a similar increase in mRNA content in the secreted exosomes.

**Figure 4:**
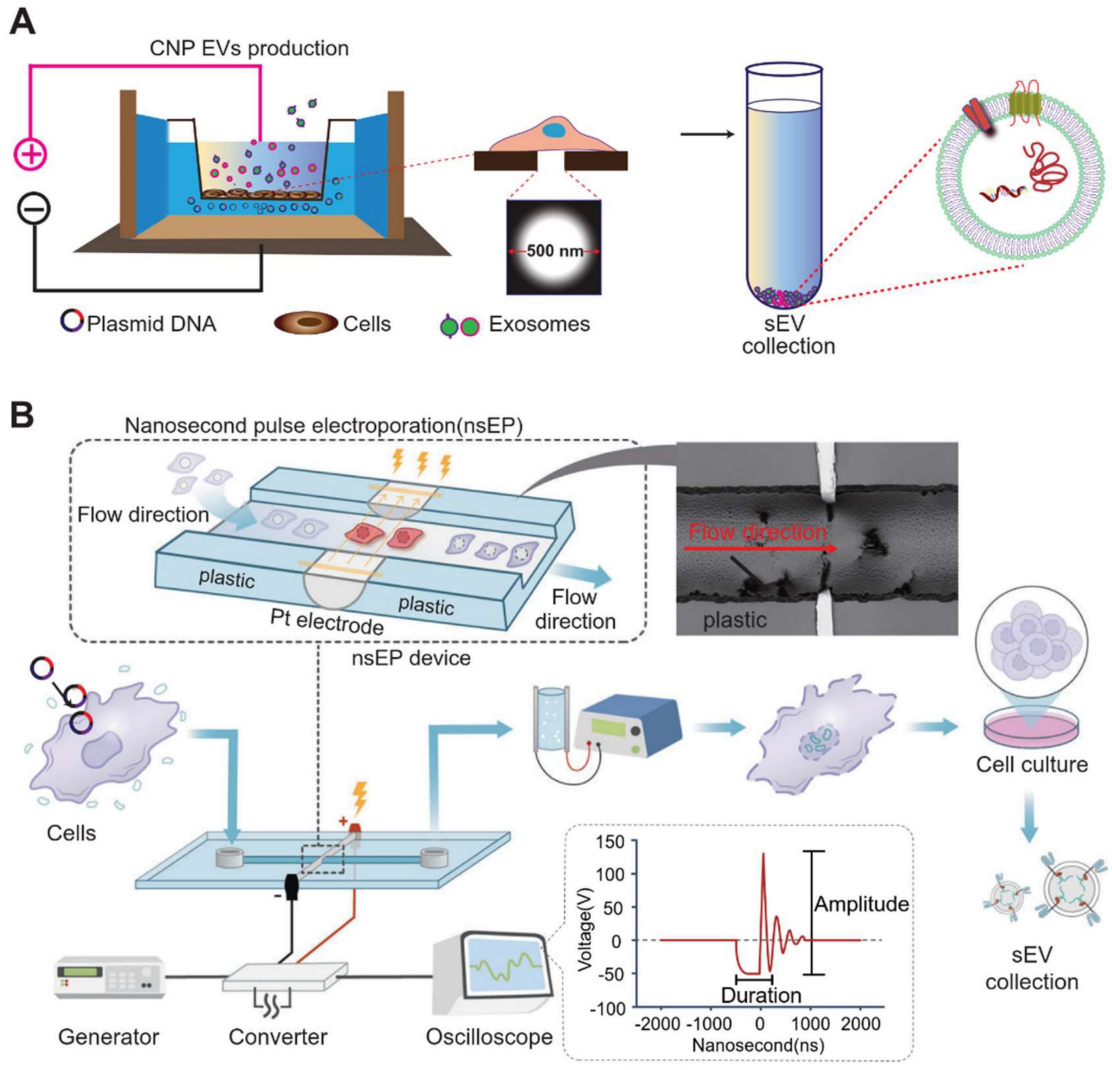
Large-scale preparation of functional exosomes loaded with mRNA using Cellular Nanopuncture and Nanosecond Pluse Electroporation (NESP) system. The figures B were adapted with permission from 98, copyright 2024 Nat Commun © Springer Nature Limited).

To further augment the amount of mRNA produced by cellular exosomes, micrometer-pore-size nanopore technology (CNP) has been developed 74. In the figure below, the cellular nanopore biochip functions specifically by utilizing a Transwell-like system in which plasmids are deposited in a pool below, while donor cells are incubated in the Transwell (**Figure 4**). By applying transient electrical stimulation to the top and bottom of the Transwell, the plasmid in the pool enters the cell through the 500 nm micropore at the bottom of the Transwell. Here, the cell transcribes the corresponding mRNA, and subsequently, exosomes release the transcribed mRNA. This cellular nanoparticulation technique produces 50-fold more exosomes than normal electroporation, and the exosomal mRNA transcripts increase by more than 103-fold in copy number. The use of biochips not only stimulates cells to secrete exosomes in large quantities but also simplifies the method of endogenous RNA loading into exosomes. A series of new cellular nanopiercing techniques now provide novel ideas and methods for the application of exosomes in mRNA delivery.

**Figure 5:**
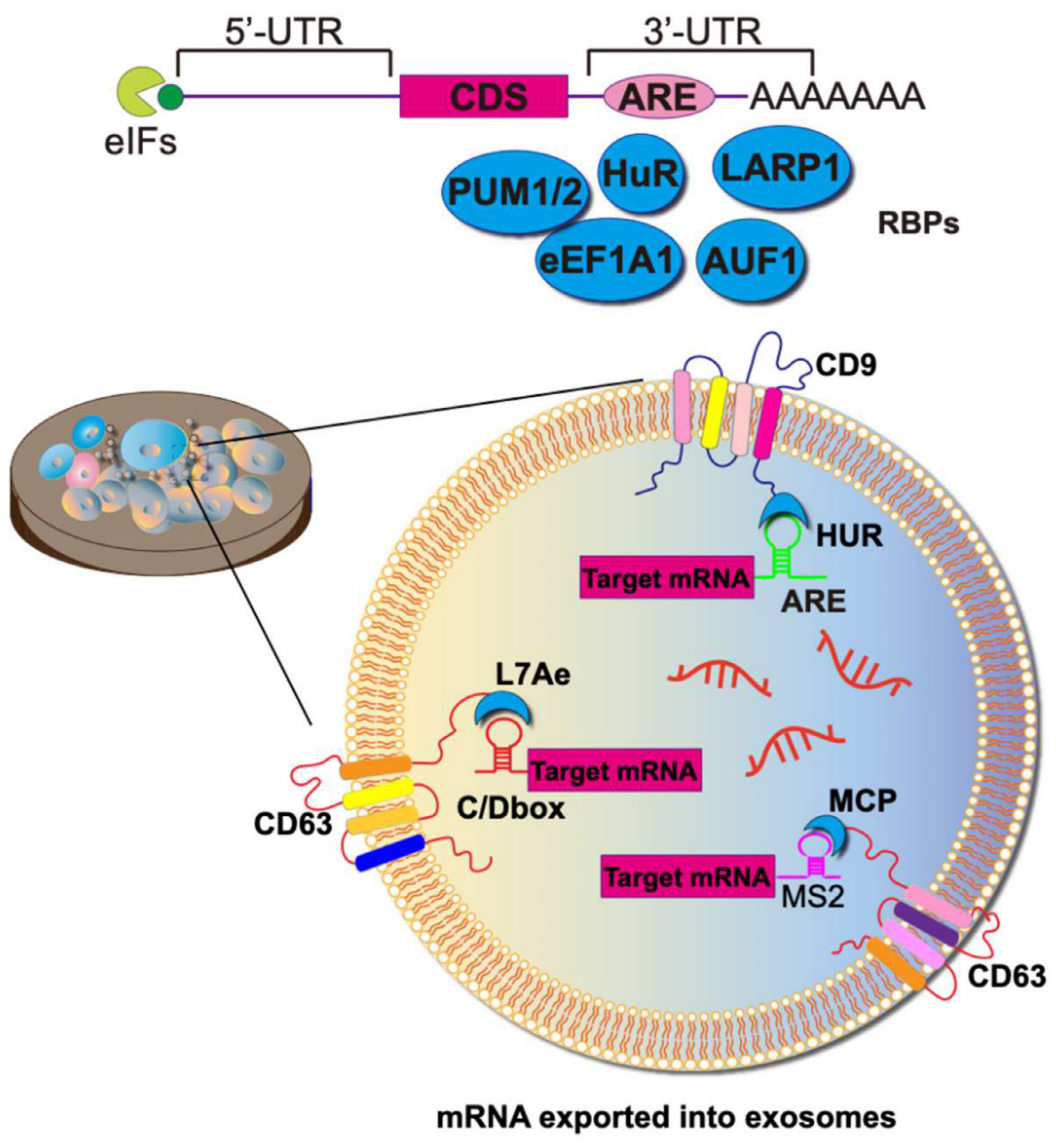
Strategies towards enriching RNAs into exosomes. To heighten the target specificity of molecular RNA export, specific RNA sequences can be encapsulated within the exosome lumen through the construction of fusion RBPs. For example, the fusion of the exosome membrane protein CD9 with the RBP HUR successfully enriches mRNAs with AU elements. Alternatively, the utilization of the phage MS2 shell protein MCP, instead of the natural RNA recognition system, increases the abundance of molecules carrying MS2-tagged target RNA. Or, an archaeal-derived L7Ae peptide was fused to the exosome marker protein CD63, which recruited and packaged C/Dbox-containing mRNAs into budding exosomes. To package RNA into the ARMMS (Arrestin domain-containing protein 1-mediated MVs), the transcription activator (Tat) protein, specifically binding to stem-loop-containing trans-activation response (TAR) element RNA, was employed.

Achieving the selective sorting of desired mRNAs into EVs remains a significant challenge. RNA-binding domains can function as crucial units responsible for mRNA sorting. In this context, mRNAs are sorted and loaded into exosomes through the binding of RNA-binding proteins (RBPs) to cis-regulatory elements present in the 3'-untranslated regions (UTRs), 5' UTRs, and coding regions. Various RBPs, such as hnRNPA2B1 75, Y-box protein 1 76, SYNCRIP 77, and the ELVA protein HuR, have been observed to be associated with RNA exportation to exosomes. The fusion expression of RBPs with exosome scaffolding proteins (e.g., CD9, CD63, CD81, Hspa8, and Lamp2b) allows for the active packaging of RNA into exosomes (**Figure 5**).

The most typical mRBPs include the MS2 bacteriophage coat protein (MCP) and the corresponding MS2 stem-loop. MCP specifically binds to RNA structures formed by the MS2 stem loops loop, as well as similar pairs like L7Ae/C/D box, Tat/TAR, HUR/AREs, and ZF/DNA aptamer (**Figure 5**). To augment the efficiency of mRNA active loading, this approach necessitates the concurrent transfection of parental cells with two plasmids. The first plasmid encodes a fusion protein comprising RBP and EV scaffolding proteins. Simultaneously, the second plasmid transcribes the mRNA of interest and incorporates a deliberately designed RBP recognition site, fostering a specific binding with the mRNA of the fusion protein. As a result, a marked increase in labeled mRNAs is discerned within EVs during their biogenesis.

HuR, an RBP that binds to adenine- and uridine-rich elements (ARE) in the 3'- UTR of the target mRNA, regulates its stability and translation. The plasmid CD9-HuR is constructed by ligating HuR to the C-terminus of CD9, and the corresponding ARE (AUUUUACCCAUUUACCCAUUUAC) loop is inserted into the 3' UTR of the CRISPR/dCas9 gene to construct CRISPR/dCas9-ARE. Subsequently, secreted CD9-HuR exosomes specifically encapsulate CRISPR/dCas9 cargoes after simultaneous transfection 78.

**Figure 6:**
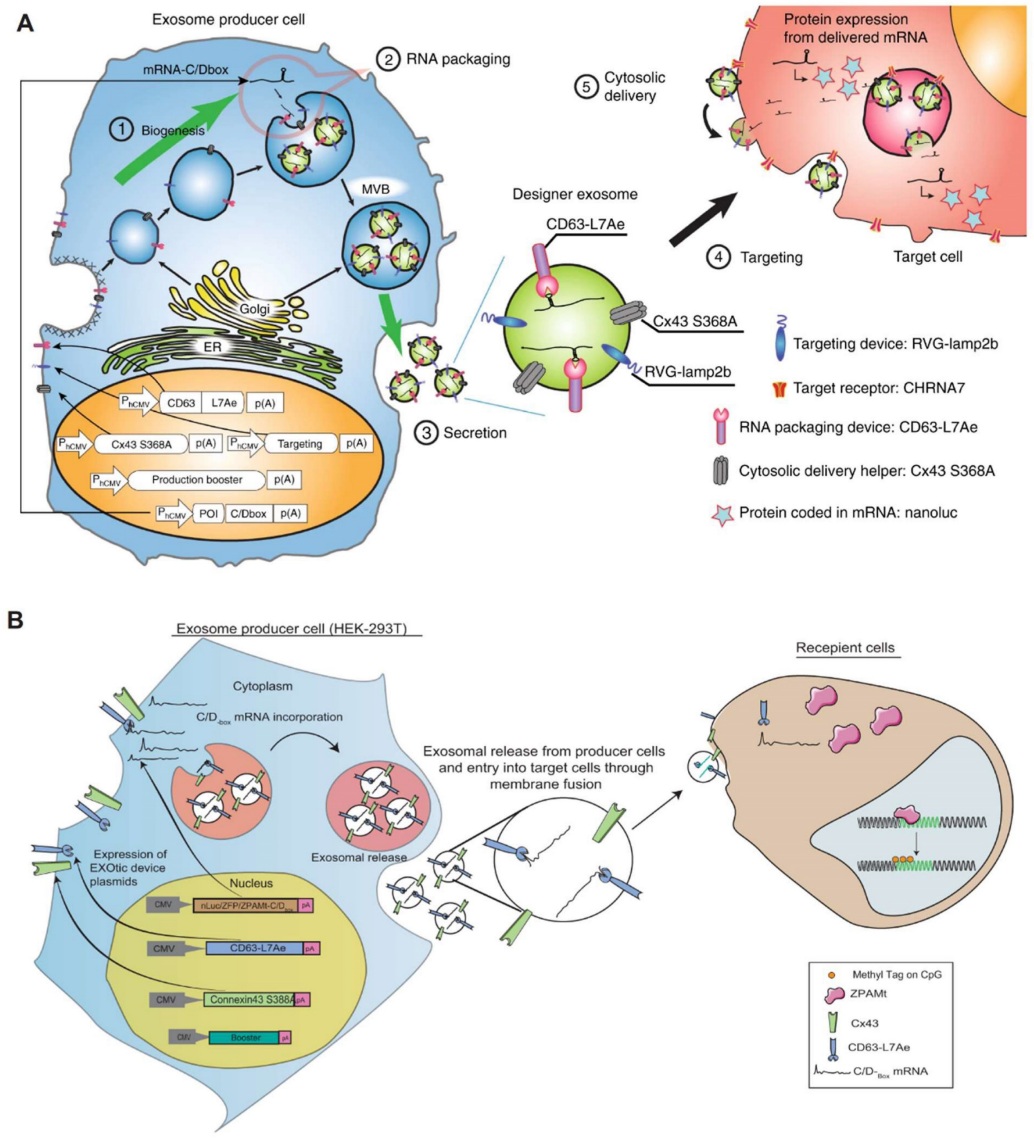
Exosomal transfer into cells (EXOtic) devices mediate mRNA delivery. In this scenario, L7Ae was linked to the C-terminus of the exosome membrane protein CD63, and a C/Dbox was inserted into the 3′-UTR of the gene of interest. This configuration facilitated the recruitment of RNA into the exosome through the interaction between the C/Dbox in its 3′-UTR and the exosome lumen-expressed L7Ae. (A) Delivery of catalase mRNA to the brain via EXOtic devices for experimental Parkinson's disease. (B) EXOtic devices for encapsulating mRNAs for zinc finger protein (ZFP-362) and a series of structural domains of DNA methyltransferase 3A to reduce the HIV-1 reservoir in HIV-1 infected cells. The figures A were adapted with permission from 80, copyright 2018 Nat Commun © Springer Nature Limited. The figures B were adapted with permission from 99, copyright 2021 Nat Commun © Springer Nature Limited).

A noteworthy active loading platform, targeted and modular EV loading (TAMEL), facilitates the directed binding of mRNA within the inner cavity of EVs (**Figure 6**). In TAMEL-producing cells, MS2 is fused to the N-terminus of the EV marker CD9 (CD90-MS2). The MS2-bound homologous stem-loop sequence is then integrated into the mRNA vector. During exocytosis, the exosome-fused MS2 protein exhibits high affinity for the stem-loop sequence-bound mRNA, efficiently sorting the mRNA cargo into the newly generated EVs 79.

Various naturally occurring RBPs can be harnessed for modular EV loading, as outlined in Table 1. For instance, the ribosomal protein L7Ae from *Archeoglobus fulgidus* exhibits an affinity for C/D box RNA secondary structures, forming the L7Ae-C/D RNA complex. Researchers fused the C-terminal inner membrane region of the exosome membrane protein CD63 with L7Ae and introduced the C/D box into the 3'-UTR of the target gene. Consequently, L7Ae on the exosome membrane effectively orchestrates the inclusion of C/D box RNA structural elements into the exosome lumen 80. Furthermore, the expression of L7Ae outside the exosome membrane allows it to bind to in vitro synthesized C/D sequence-labeled mRNA antigens, facilitating the delivery of mRNA to dendritic cells via OMVs 81.

Another innovative approach involves a DNA aptamer designed to act as a connecting bridge for loading the mRNA of interest into exosomes. In this scenario, one of the exosome surface markers, CD9, is fused with a zinc finger, enabling specific binding to the DNA aptamer. The single-stranded portion of the DNA aptamer recognizes the AUG region of the target mRNA, preventing mRNA translation and ribosome assembly. Theoretically, the mRNA of interest would be recruited to EVs by the corresponding DNA adaptor after DNA transcription. To validate this hypothesis, they conducted an experiment with GFP mRNA and demonstrated that mRNA for GFP is enriched in CD9-ZF-engineered EVs 82.

The predominant form of RNA in secreted exosomes is small RNAs, posing a technical challenge for passive preloading methods to encapsulate large mRNAs into the nanosized exosomes. In the active loading strategy, the loading efficiency decreases with the growing size of the mRNA. Furthermore, endogenous loading processes lead to the packaging of mRNA-encoded proteins into EVs, which is often undesirable.

MVs offer a larger capacity compared to exosomes. Consequently, studies have employed MVs to load mRNA macromolecules. In a system designed for specific mRNA loading, researchers have efficiently generated EVs loaded with RNA cargo by incorporating the Tat polypeptide from HIV into ARRDC1 and its RNA-binding element (the transcription-activation response, TAR), which is engineered to package RNA cargo. This strategy successfully delivers p53 mRNA in vivo, with translation occurring in recipient cells upon uptake 100.

In addition to using viral components, alternative approaches have leveraged "zipper code" sequences typically found in the 3′-UTR of RNA cargo. These sequences can be recognized by specific RBPs associated with membrane target proteins or MVB target proteins. Results indicate that the Zipcode structure, specifically the CUGCC core of the 3′-UTR and a miRNA binding site, plays a crucial role in enhancing mRNA entry into MV. Subsequently, HEK-293T cells transfected with Zipcode fused to the EGFP stem-loop structure demonstrate a twofold increase in the amount of EGFP mRNA in MV 101.

### 4.2 Post-loading methods

Post-loading methods, also referred to as post-isolation or exogenous loading methods, involve the in vitro loading of synthesized mRNAs into isolated CDVs primarily through chemical or physical means. Electroporation is a widely used technique for loading various molecules, including siRNAs, miRNAs, and mRNAs, into purified CDVs. For instance, approximately one-fifth of Cas9 mRNA can be loaded into erythrocyte-derived EVs through electroporation.

In a groundbreaking study, micro- and nanofluidic technologies are employed to apply mechanical compression force and fluid lateral shear force directly to the exosome vesicle membrane. This approach generates transient nanopores on the membrane surface without disrupting the lipid membrane structure, allowing for the non-invasive loading of therapeutic "cargo" molecules from the surrounding solution into the exosome. This method holds promise for efficient mRNA cargo loading into exosomes in the future 102.

Additionally, chemical transfection has been utilized to incubate mRNA into CDVs, with commercial loading reagents such as REG1 or Exo-Fect proving effective. Despite achieving high loading efficiency, challenges arise in purifying CDVs from excess transfectants, potentially leading to cytotoxicity. Other physical methods, including sonication, extrusion, and repeated freezing and thawing, are also commonly employed for active exosome loading. These methods typically involve disrupting and subsequently restoring the integrity of the EV membrane for drug encapsulation. However, such loading approaches may be less efficient, possibly due to the formation of aggregates during the physical process. Each method comes with its own set of advantages and limitations. It is crucial to note that violent loading procedures that may compromise EV integrity or induce aggregation should be avoided.

## 5. The application potential of mRNA-loaded CDVs

In comparison to traditional small molecule and antibody drugs, mRNA has emerged as a novel class of drugs, offering advantages in target selection and drug design that significantly advance the drug development process. mRNA drugs predominantly function in the cytoplasm, minimizing the risk of integration into the genome and thereby avoiding potential gene mutations and cumulative toxicity. The versatility of mRNA allows for encoding proteins or peptides to facilitate transient expression in target cells, enabling protein replacement therapy to address deficiencies. Additionally, mRNA has shown promise in vaccination for antigen presentation, contributing to cancer immunotherapy and infectious disease treatments. The use of mRNA encoding cas9 also allows for effective gene editing, offering a broad spectrum for treating various diseases.

Following the success of mRNA technology in COVID-19 vaccines, its application in other fields has garnered widespread attention. Over 100 clinical trials for mRNA vaccines have been licensed, and six mRNA therapies targeting cancers are currently in phase II clinical trials. Therefore, there is significant potential for the clinical application of mRNA therapeutics in diverse areas.

Nanoscale delivery systems play a crucial role in the development of mRNA vaccines and related therapies. The majority of mRNA reagents are delivered by nanocarriers formed from lipid materials. Exosomes, vital in nucleic acid cargo transportation systems, play a particularly important role. In comparison to synthetic nanoparticles, natural cell-derived exosomes demonstrate excellent biocompatibility, low immunogenicity, and small size, which hinders clearance by mononuclear phagocytes. Exosomes exhibit high permeability and retention effects, facilitating drug accumulation at the target site (**Figure 3**). When employed as mRNA delivery systems, CDVs, especially exosomes, show great potential in various biomedical applications, including oncology, central nervous system (CNS) disorders, obesity, and anti-inflammation (**Table 2**).

### 5.1 CDV-based mRNA therapy to the central nervous system disorders

CDVs have the inherent ability to traverse physiological barriers, including the BBB, positioning them as promising carriers for treating various brain diseases. Specifically, exosomes modified with RVG peptides on their membrane present an effective means to target neuronal cells, facilitating drug delivery to the CNS upon tail vein injection. In a preliminary study, researchers have engineered RVG peptides on the surface of exosomes for neuronal targeting, concurrently loading mRNA of nerve growth factor (NGF) through plasmid transfection. The resulting product, named NGF@ExoRVG, allows NGF to be exported into RVG-Lamp2b-engineered exosomes. Following tail vein injection, NGF@ExoRVG successfully reaches the ischemic cerebral cortex. The encapsulated NGF mRNA is translated, giving rise to new NGF proteins directly within the recipient cells.

Functional outcomes from this innovative approach indicate that exosomal delivery of NGF leads to the remodeling of microglia polarization, resulting in reduced inflammation, an increased number of neuroblastoma cell markers, and enhanced neuroregeneration in ischemic mice. These effects contribute to alleviating ischemic brain damage during stroke 108. This novel and straightforward strategy of exosomal mRNA delivery provides valuable insights into the clinical application of other neurotrophic factors for the treatment of CNS diseases.

Researchers have devised and implemented an ingenious exosome-producing gene system known as EXOtic. This comprehensive system comprises four key components: an exosome production booster, a specific mRNA packaging device (CD63-L7Ae, C/Dbox bound to RNA), a cytoplasmic delivery helper, and a brain-targeting module (RVG-Lamp2b). When these modules are intentionally overexpressed in mammalian cells, they orchestrate the production of exosomes. This EXOtic system exhibits impressive capabilities, particularly in loading catalase mRNA into exosomes and facilitating its transport to the brain. This tailored approach effectively diminishes neuroinflammation, showcasing notable efficacy in mitigating neuroinflammatory responses in both a rat model of Parkinson's disease and in lipopolysaccharide (LPS)-induced neuroinflammation 80. An application of the EXOtic system involves the implantation of modified exosome-producing cells into living mice. This in situ approach allows for the production and delivery of therapeutic mRNA-containing exosomes within the living organism. In a model of Parkinson's disease, this method demonstrates its potential by rescuing neuronal cell death in specific regions of the brain, offering a promising avenue for the treatment of neurodegenerative conditions.

To achieve efficient targeted delivery to neurons, Gu *et al*. found that bone marrow (BM) dendritic cells (DCs) and macrophages (MΦs) were most effective for penetrating the blood-brain barrier, with unique selective accumulation in neuroinflammatory regions. In addition, the lumen of their EVs was doped with Arc, a retrovirus-like protein coat naturally occurring in the human brain, to obtain the engineered retrotransposon Arc EVs (eraEVs). The addition of the Arc 5' UTR (A5U) to the mRNA sequence stabilized the interaction with the Arc protein, which increased the loading efficiency of the eraEVs for mRNA 120. This approach showed high efficiency and safety in both *in vitro* and *in vivo* experiments, providing the ability to deliver mRNAs for the treatment of ischemic myocardial infarction, ischemic stroke, and brain tumors.

### 5.2 CDV-based mRNA delivery systems for cancer therapies

Engineered CDVs have been found to be useful in cancer therapies. Researchers studied the therapeutic potential of exosomes modified with glioma-targeting peptides (Exo-T) for delivering PTEN mRNA against gliomas. The study's outcomes reveal that intravenous injection of Exo-T into mice of a glioma model targeted the mRNA to glioma cells in the brain through the BBB. This treatment resulted in the restoration of tumor suppressive capacity, leading to an intensified inhibitory effect on tumor growth and an improved survival rate for mice with U87 or GL261 gliomas 74.

Plasmid transfection or lentiviral infection are viable methods of encapsulating mRNA in CDVs. One such case involves stable transfection of 293-T cells with a plasmid encoding the suicide-encoded cytosine deaminase (CD)-uracil phosphoribosyltransferase (UPRT). The collected genetically engineered MVs can effectively deliver CD-UPRT mRNA into target tumor cells, showcasing a promising avenue for cancer therapy 112. In another study, tumor tissues treated with CD-UPRT mRNA exosomes exhibited elevated levels of the CD-UPRT enzyme. This enzyme converts the chemotherapeutic agent 5-fluorocytosine (5-FC) to 5-fluorodeoxyuridine monophosphate (5-FDUP), inducing apoptosis in tumor cells. In vivo experimental results demonstrated significant inhibition of schwannoma tumor growth and reduced tumor volume 113. Researchers also employed the "zipcode" technique to insert the 3′-UTR of the HchrR6 gene into two tandem copies of the zipcode-like nucleotide sequence. This facilitated the loading of mRNA cargo into CDVs. By expressing anti-HER2 scFv antibody on the exosomal membrane, they achieved specific targeting of HER2-positive cancer cells. Co-administration of HChrR6 mRNA-exosomes loaded with CNOB resulted in the specific targeting of HER2+ cells and effectively inhibits the growth of HER2+ breast cancer tumors *in vivo* 114. Another study aimed for a safer delivery method for HChrR6 mRNA to treat breast cancer. In this approach, in vitro transcribed (IVT) HChrR6 mRNA was packaged into exosomes, avoiding horizontal gene transfer associated with plasmid DNA transfection. The HChrR6 protein could convert the prodrug CNOB into the cytotoxic drug MCHB. Exosome-mediated CNOB/ChrR6 therapeutics proved effective in tumor suppression 122. Loading IL-12 mRNA into extracellular vesicles using the inhalation route can be used to achieve more precise delivery to the tumor microenvironment. Liu et al. enhanced the distribution of IL-12 in the lung tumor microenvironment while minimizing systemic toxicity. The results of this study suggested that these inhaled IL-12 exosomes promoted IFNγ-mediated immune activation, immune memory formation, and initiation of tumor-specific T cells, ultimately suppressing lung tumors and tumor reinvasion 118. In another study, researchers showed that CD9-HuR exosomes could enrich the functional CRISPR/dCas9 when the RNAs were engineered to have AU-rich elements 78. Moreover, human erythrocytes serve as exosomal donors for RNA therapy, with Cas9 mRNA and sgRNA targeting the human mir-125b-2 gene locus loaded inside, which significantly inhibited the expression of miR-125a and miR-125b, demonstrating potent in vivo therapeutic efficacy for acute myeloid leukemia (AML) 123.

Additionally, OMVs, naturally secreted lipid bilayer nanostructures by Gram-negative bacteria, were surface modified with the RBP L7Ae. This platform, OMV-LL-mRNA, was used to deliver box C/D sequence-tagged ADPGK mRNA antigens to dendritic cells, which mediated endosomal escape through listeriolysin O (LLO), and significantly inhibited melanoma evolution after subcutaneous injection. The OMV-based nanocarrier platform, distinct from lipid nanoparticle (LNP)-mRNA vaccines, serves as a natural immunotherapy carrier with the potential to improve the tumor immunosuppressive microenvironment and act as a versatile drug delivery vehicle, providing a "plug and play" technology for the rapid preparation of personalized mRNA tumor vaccines 121, 124.

### 5.3 CDV-based mRNA in regeneration medicine

Skin atrophy, marked by the irreversible loss of collagen, is a prominent characteristic of skin aging. Various approaches have been explored to address collagen loss and halt skin aging, including laser treatment and antioxidants with vitamin A-like agents. However, these existing pharmacological techniques fall short of achieving long-term endogenous collagen replacement, leading to consistent skin firmness and elasticity over time. In a recent study, researchers developed an exosome-based therapy utilizing COL1A1 mRNA for the anti-aging treatment of photoaged skin 125. The mRNA-loaded EVs were efficiently delivered into the dermis via hyaluronic acid microneedle patches, promoting the production of collagen lost due to aging in recipient skin cells. Consequently, EV-mediated mRNA for collagen protein replacement therapy restored skin elasticity and firmness in a collagen-deficient, photoaging-induced animal model.

### 5.4 CDV-based mRNA vaccines for infectious diseases

The onset of the COVID-19 pandemic has ushered humanity into the RNA era, with LNPs widely employed for RNA vaccine delivery. Notable examples include BNT162b1 (BioNTech and Pfizer), the ARCoV mRNA vaccine, and mRNA-1273 (Moderna), all of which have shown effectiveness in human trials 126-129.

**Figure 7:**
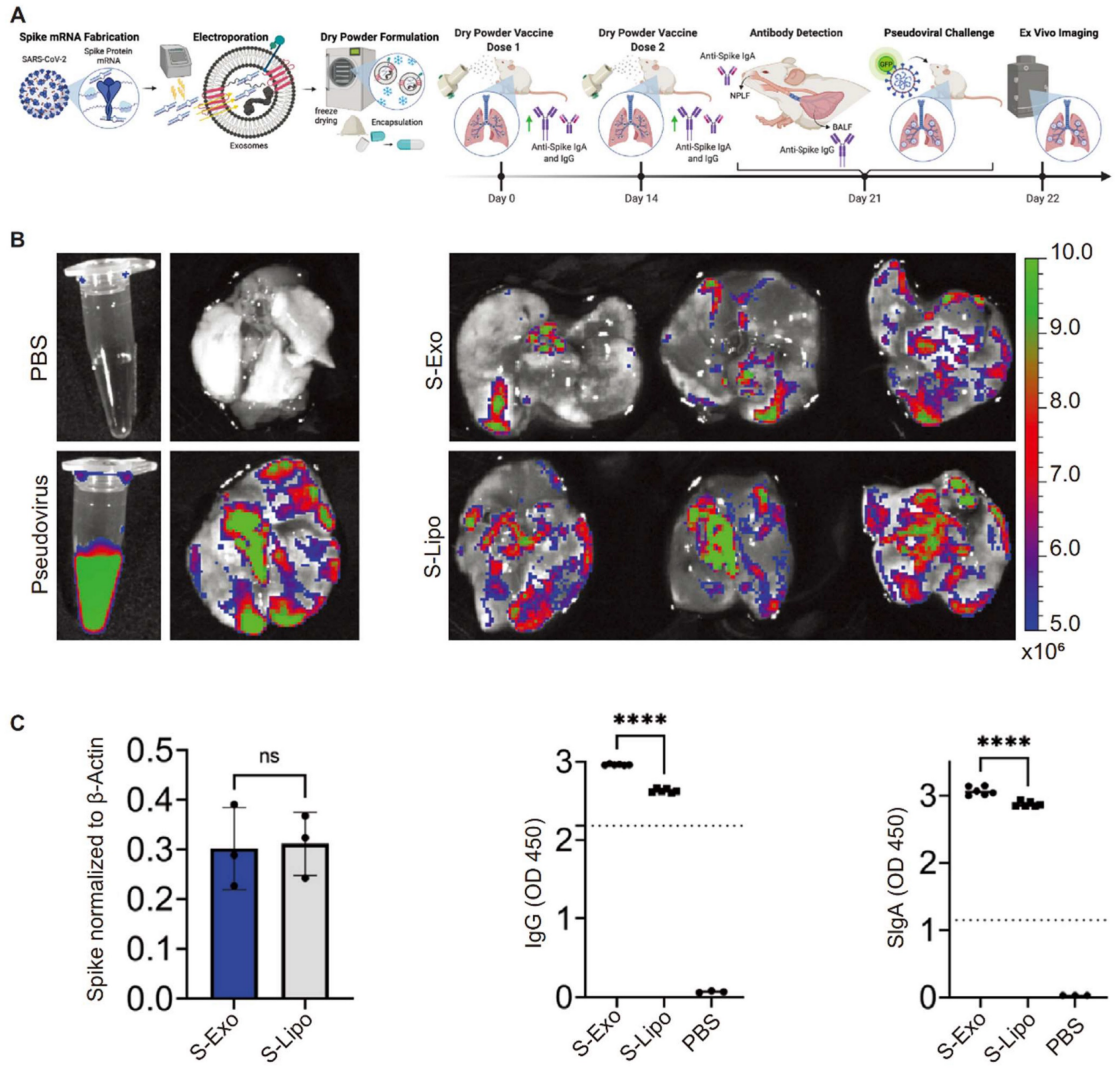
Lung-derived extracellular vesicles or exosomes to package mRNAs that activate humoral immunity as neo-crown vaccines. The figures (A-C) were adapted with permission from 119, copyright 2022 Matter © Elsevier, Inc.

Recently, an mRNA vaccine against COVID-19 was developed by electroporating mRNA and encoding SARS-CoV-2 spikes and nucleocapsid proteins into lung-derived exosomes. Distinct from liposome-based vaccines, this vaccine, when inhaled into mice via dry powder, triggered humoral immune responses and activated mucosal immunity. Lung-derived EVs or exosomes (lung-Exos) retained functionality even after a month of storage at room temperature. In comparison to standard synthetic nanoliposomes, lung-Exos loaded with mRNA exhibited better distribution in respiratory and alveolar epithelial cells of African green monkeys, promoting immunoglobulin G (IgG) and secretory IgA (SIgA) responses more effectively than synthetic liposomes 119 (**Figure 7**). These findings suggest that exosome-based inhaled mRNA delivery systems surpass synthetic liposomes.

### 5.5 CDV-mediated delivery of mRNA for atherosclerosis treatment

In contrast to vaccination and tumor immunotherapy, protein replacement therapy using mRNA is currently in preclinical development. This therapeutic approach involves introducing mRNA to produce therapeutically functional proteins, replacing mutated or supplementing deficient proteins. This innovative strategy has shown promise in various medical fields, including inherited metabolic diseases, solid tumors, and cardiovascular diseases. A notable application involves the low-density lipoprotein receptor (LDLR), which is widely distributed on the surface of cell membranes in tissues throughout the body, including liver and arterial wall cells. LDLR facilitates the uptake of extracellular cholesterol into cells, thereby reducing LDL cholesterol in plasma and preventing atherosclerosis resulting from lipid deposition in the vascular endothelium. In a recent study, exosomes encapsulating Ldlr mRNA were produced by overexpressing the Ldlr gene in donor hepatocytes. Using the Ldlr-/- mouse model, the study demonstrated that exosome-mediated delivery of Ldlr mRNA rapidly restored LDLR expression. This led to a reduction in high serum cholesterol levels and the reversal of phenotypes such as steatosis and atherosclerosis 107. In clinical trials, good manufacturing practice (GMP)-compatible bone marrow mesenchymal stem cells (MSCs) were manufactured to generate Ldlr mRNA-enriched exosomes. This approach was poised to provide a new mRNA therapeutic strategy for patients with homozygous familial hypercholesterolemia [Clinical Trial: NCT05043181].

In another mRNA therapeutic study for atherosclerosis, IL-10 mRNA was encapsulated into exosomes through transient transfection in parental cells. To achieve miR-155-responsive IL-10 translational activation, researchers replaced the miR-122 recognition region of the hepatitis C virus internal ribosome entry site (HCV-IRES) with a miR-155 recognition site, ligated it downstream to the coding sequence (CDS) of IL-10, and engineered exosomes containing IL-10 mRNA. These engineered exosomes selectively delivered IL-10 mRNA into the inflammatory milieu of atherosclerotic plaques, amplifying the anti-inflammatory effects of IL-10 mRNA therapeutics. The resulting anti-inflammatory effects alleviated atherosclerosis in ApoE-/- mice, reducing the size of atherosclerotic plaques and lesions 106. These efforts held promise for treating atherosclerosis and represent a positive step toward clinically translating mRNAs into new therapeutic options for related cardiovascular diseases.

### 5.6 Other applications

Moreover, a recent article reported the sorting of DNA aptamer-IL-10 mRNA complexes into CD9-ZF-engineered EVs for treating inflammatory bowel disease 54. Systemic injection of exosomes containing IL-10 mRNA successfully penetrated local mucosal tissues, inhibiting colon length shortening and attenuating inflammatory responses in a dextran sulfate sodium (DSS)-induced mouse model 82. Additionally, specific delivery of Pgc1α mRNA to adipose tissue through this exosome delivery strategy promotes white fat browning, offering a potential treatment for obesity 103. To address off-target effects, tissue-specific translation-activated engineered mRNAs, modified with miRNA recognition sites, were simultaneously encapsulated within exosomes. Engineered exosomes delivering mRNAs to adipose tissue cells were activated by adipose-specific miRNAs, inducing safe and effective adipose browning to combat obesity 103.

In another approach, ultrasound was utilized to assist exosome delivery. Bone morphogenetic protein 7 (BMP7), a cytokine with multiple functions, was shown to drive the differentiation of brown adipocytes in adipose tissue. Exosomes delivering Bmp7 mRNA to adipose tissue under ultrasound promoted the localized conversion of white to brown fat, increasing energy metabolism for obesity treatment. Moreover, ExoCP05 peptide-modified exosomes, loaded with an ultrasound sensitizer (Ce6), were designed to prolong blood circulation time. Under ultrasound, Ce6 generated reactive oxygen species (ROS), cleaving the TK bond and releasing Bmp-7 exosomes from the PEG protective layer. This construction of ExoCP05-TK-mPEG provided an advanced platform for ultrasound-assisted mRNA delivery strategies 105.

Collectively, the orchestrated exosomal processing of upstream transcripts of interest facilitated the inception of a groundbreaking, tissue-specific induced mRNA expression. This not only introduces innovative concepts for enhancing therapeutic efficacy but also holds the potential to mitigate off-target effects. In summary, these investigations delineate a compelling approach for encapsulating therapeutic mRNAs within CDVs, thereby offering a promising way for protein replacement therapy utilizing mRNA.

## 6. Conclusion and prospects

mRNA-based therapeutics have been realized, marked by two mRNA vaccines for combating COVID-19. Nevertheless, the utility of mRNAs is still constrained by challenges, such as stability, delivery efficiency, and heightened immunogenicity. While LNPs are the favorite carriers for mRNA vaccines and have seen extensive development, emerging evidence suggests that exosomes may be an alternative option 130, 131. Exosomes exhibit numerous advantages as mRNA delivery vectors, characterized by commendable biocompatibility, low immunogenicity, and the capacity to traverse biological barriers, including BBB. However, CDVs for mRNA delivery still need to overcome various issues, such as standardized protocols for large-scale production, the purity of the active substance, ideal dosage, immunogenicity, and effective loading of the therapeutics.

### 6.1 Scalability in manufacturing

Bringing CDV-based mRNA nanodrugs from the laboratory to the marketplace requires a simple, scalable, and reproducible manufacturing protocol. However, many CDVs contain heterogeneous components with different cellular functions in terms of structure and composition. To address these major challenges, research efforts have focused on two main directions: selecting suitable donor cells to obtain CDVs and producing and isolating high-purity CDVs on a large scale.

Large-scale production and purification of CDVs, coupled with the implementation of standardized and efficient mRNA loading methods, stand out as significant hurdles for the clinical use of CDVs. Investigations have been conducted to enhance exosome production by downregulating genes that impede exosome biogenesis or introducing erythrocyte membrane components into the culture medium. Notably, the depletion of Rab4 among candidate genes has proven effective in promoting exosome biosynthesis. Additionally, the supplementation of red cell membrane particles (RCMPs) in the culture medium has demonstrated a further augmentation of exosome production. Currently, there are no unique and ideal purification techniques for separating and isolating high-purity CDVs, and there is a need to integrate these synthetic procedures into equipment or pipelines for the continuous production of CDVs. Recently, there has been a growing demand for reproducible large-scale CDV purification, with tangential flow filtration (TFF) methods emerging as a focal point. Consequently, developing standardized and reliable CDV purification methods aligned with GMP guidelines becomes imperative.

### 6.2 Targeted and stimuli-responsive mRNA delivery

The advancement of targeted delivery systems and steadfast platforms for mRNA emerges as a pivotal technological frontier in mRNA therapy. In the realm of targeted delivery, there exists an imperative demand for the creation of delivery vehicles capable of precise mRNA transport to designated organs and cells. Despite the widespread development of nucleic acid nanocarriers, a predominant challenge remains in their propensity to nonspecifically deliver mRNA, primarily to the liver and spleen upon systemic administration. This significantly curtails the clinical applicability of mRNA drugs. Engineering methods, such as genetic engineering or chemical modification, can install targeting moieties on the surface of CDVs, but they must preserve the morphology and size of CDVs 47, 132-135.

Achieving tissue-specific expression of delivery genes minimizes off-target effects. Traditionally, tissue-specific promoters are employed for conditional gene expression, albeit controlling gene expression solely at the transcriptional level when delivering DNA. In contrast, achieving tissue-specific control of gene expression at the translational level requires the delivered RNA to act exclusively in the target tissue. For instance, the liver-specific miRNA-122 (miR-122) recognizes conserved sites in the IRES region at the 5′ end of the HCV genome, thereby stimulating IRES-mediated translation. 136 Notably, adipose tissue-specific overexpression of miR-148a activates the translational activity of exosome-delivered miR-148a-IRES-mRNA in a sequence-dependent manner 103.

Identifying cell type-specific patterns in miRNA expression provides a way to achieve tissue specificity. Furthermore, strategies integrating responsive elements onto the exosome surface, such as pH-driven, ultrasound, and magnetically guided mechanisms, also provide possible ways to realize the localized release of CDVs 103. Stimuli-responsive exosome delivery systems may realize gene expression in specific receptor cells while substantially mitigating off-target effects in gene therapy.

**Figure 8:**
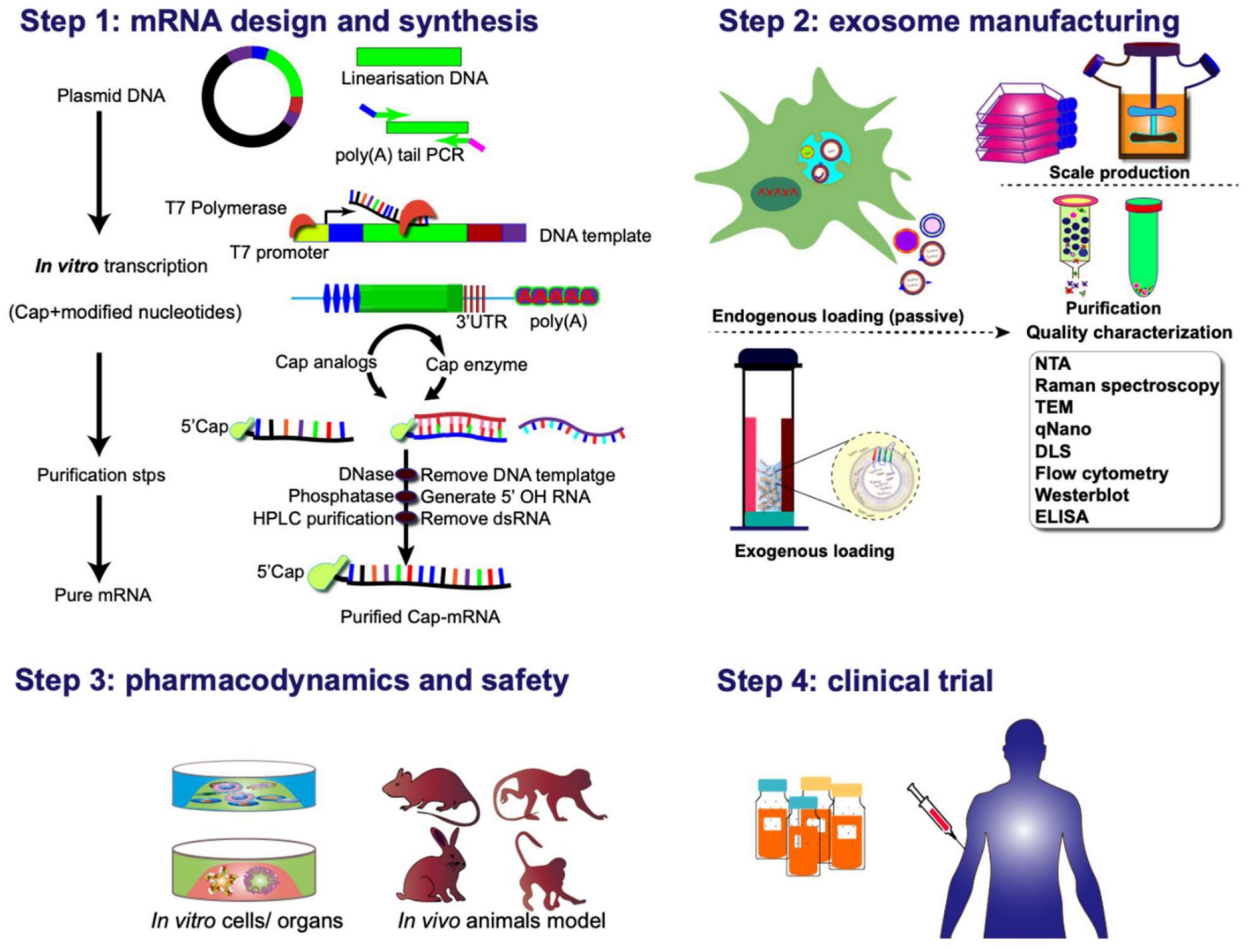
Clinical pipeline for exosome-based mRNA therapies. The process commences with the thoughtful design and incorporation of protein-coding sequences into plasmid DNA constructs. Following this, the plasmid DNA is linearized, subjected to reverse transcription into mRNA in vitro, and subsequently undergoes nucleoside modification and purification before being encapsulated into exosomal vectors. An alternative pathway involves the intracellular synthesis of mRNA-exosome nanostructures through the active loading of mRNA during exosome biogenesis. This intricate process sets the stage for subsequent evaluations encompassing pharmacodynamics, pharmacokinetics, *in vivo* and *in vitro* safety assessments, as well as considerations for manufacturing and clinical trials. These comprehensive evaluations form the foundation for advancing the development of exosomal-mRNA, ensuring a thorough understanding of its efficacy, safety profile, and overall feasibility for clinical application.

Various strategies can be employed for clinical applications of exosome-mediated mRNA delivery. One approach involves culturing the patient's cells and genetically modifying them according to the specific requirements for targeting and delivering therapeutic proteins/ribonucleic acid. Subsequently, exosomes isolated from the cell culture are injected into patients. The second strategy adopts an off-the-shelf cell therapy model, wherein mRNA-loaded exosomes are delivered to isolated cells, which are then reintroduced into the organism. Or, *in vivo* genetic modification of cells to produce conditional therapeutic CDVs can be employed. The rapid and automated manufacturing of clinical-grade mRNA preparations for encoding diverse proteins in a scalable, exosome-encapsulated form, can be achieved with a few simple steps (**Figure 8**). These hold promise for addressing the unmet clinical needs of numerous patients or those undergoing treatments with inherent challenges.

## Figures and Tables

**Table 1:** RNA binding proteins and their corresponding loops.

RNA binding protein	Original	RNA Ioop	RNA Ioop sequence element	Function	Application	Binding efficiency	References
**HuR**	Prokaryotes and eukaryotes	NA	NA	Controls mRNA stability and turnover	Targeting HuR for cancer therapeutics, immune regulator	0.05 nm	83
**TISS11**	Eukaryotes	Poly(A) tails	NA	Promote mRNA degradation	NA	NA	84
**QKI**	Eukaryotes	KH domain	NA	Controls mRNA stability and translation	Positive effects in the treatment of neurological disorders, bone metabolism, cardiovascular system, and cancer	NA	85
**hnRNPs**	Eukaryotes	NA	NA	Pack and stability freshly transcribed pre-mRNAs	Involved in cancer and neurodegenerative disease	NA	86
**NF90**	Mammalian	3'UTR	NA	Regulating mRNA turnover and translation	Proangiogenic activity	NA	87
**YB1**	Eukaryotes	Alanine/proline-rich N-terminal domain, a cold shock domain and a C-terminal domain	NA	mRNA splicing, stability, and translation	Biomarker of prognosis and malignancy	NA	88
**RIG-I**	Mammalian	A middle DExD/H-box helicase domain	NA	Receptor for viral dsRNA.	Inhibit tumor growth	NA	89
**U1A**	Eukaryotes	Amino-terminal RNA-binding domain of U1 SnRNP A	RNA hairpin	Promotion of mRNA expression	NA	10 pM	90
**Lambda N**	λphage	Lambda box-B RNA sequence	BOX-B	Promote mRNA transcription	NA	1.9 nM	91
**P22N**	P22 phage	3-out/4-out GNRA-like pentaloop	BOX-B	Promote mRNA transcription	NA	5 pM	92
**La**	Eukaryotes	NA	NA	Regulator mRNA stability	Targeting La for cancer therapeutics	NA	93
**MS2**	MS2 phage	MS2-binding site	BOX-B	Determine mRNA stability	Diagnostic markers for infectious diseases	NA	94
**Qβ**	Phage	QβStem-loop	NA	Alter mRNA stability	Biochemical markers for the prognosis	NA	95
**PP7**	PP7 phage	PP7 RNA sequence	Specific RNA-binding structural domains	Controls mRNA stability	Diagnostic markers for infectious diseases	NA	96
**NRE**	Eukaryotes	Nanos response Elements	NA	Promotes mRNA degradation	Diagnosis and treatment of cancer	NA	97

**Table 2:** Biomimetic cellular membrane-derived nanocarriers for mRNA delivery

Source cell	Nanoparticle	mRNA	Loading method	Target cell	Effect	Refs.
**HEK293T**	Exosomes	PGC1α	Plasmid transfection	Visceral adipose tissue	Induced fat browning and holds promise for obesity therapy	103
**293F**	Exosomes	SARS-CoV-2 spike Nucleocapsid proteins	Exosomes-mRNA lipids co-incubation	T cells	Antigen-specific CD4^+^ and CD8^+^ T-cell responses, against COVID-19/severe acute respiratory syndrome coronavirus 2 (SARS-CoV-2)	104
**HEK293T**	Exosomes	BMP-7	Plasmid transfection with smart exosome-based system	Omental adipose tissue (OAT)	Induced local white adipose tissue browning	105
**HEK293T**	Exosomes	IL-10	Plasmid transfection	Macrophages	Alleviated the atherosclerosis	106
**AML12 (alpha mouse** **liver) cells**	Exosomes	Ldlr	Plasmid transfection	Liver	Decreased lipid deposition in the liver and lowered the serum LDL-cholesterol level	107
**HEK293T**	Exosomes	Nerve growth factor (NGF)	Plasmid transfection	Neuron of ischemic cortex	Reduced inflammation by reshaping microglia polarization, promoted cell survival	108
**Adipose-derived stem cell (ADSC)**	Exosomes	Neurotrophin-3 (NT-3)	Plasmid transfection and virus infection	Schwann cells (SCs)	Promoted nerve regeneration and improved the function recovery of gastrocnemius muscles	109
**Milk**	Exosomes	SARS-CoV-2 RBD	Exosomes-mRNA lipids co-incubation	NA	Introduced immunity against SARS-CoV-2	110
**Monocyte**	Exosomes	Sox9	Plasmid transfection	Chondrocytes	Promoted the secretion of extracellular matrix components proteoglycan and type II collagen	111
**Reactive astrocyte**	Exosomes	O6-alkylguanine DNA alkyltransferase (MGMT)	Plasmid transfection	Glioma cells	Protected MGMT-negative glioma cells from TMZ-induced apoptosis	112
**HEK293T**	MVs	Uracil phosphoribosyltransferase (UPRT)	Plasmid transfection	Schwannomas	Inhibition of schwannoma tumor growth	113
**293FT**	EVs	HChrR6	Plasmid transfection	HER2+ cells	Specifically killed HER2+ cells and caused breast tumor growth arrest	114
**HEK293T**	Exosome-liposome hybrid nanoparticles	m6A eraser ALKBH5	Freeze-thaw method and the lipid membrane fusion	Colorectal cancer cells	Inhibited the progression of CRC in preclinical tumor models by modulating the ALKBH5/JMJD8/PKM2 axis and inhibiting glycolysis	115
**HEK-293T (EXOtic** **devices)**	Exosomes	Catalase	Plasmid transfection	Neuronal cells	Protection against neurotoxicity and neuroinflammation	80
**Red blood cell**	EVs	Cas9	Electroporation	Leukemia cells	Gene knockout with CRISPR-Cas9 genome editing in leukemia and breast cancer cells *in vitro* and* in vivo*	116
**SK-LU-1 Lung adenocarcinoma cells**	Exosomes	p53	Plasmid transfection	Macrophages	Mediated macrophage repolarization towards a more pro-inflammatory/antitumor M1 phenotype	117
**HEK293T**	Exosomes	CRISPR/dCas9	CD9-HuR and AU rich elements	THP1 cells	Effectively deliver CRISPR/Cas9 system and reduced C/ebpα expression in the liver	78
**HEK 293T**	Exosomes	IL-12	Electroporation	Lung cancer	Inhaled IL-12-Exo promoted IFNγ-mediated immune activation and tumor-suppressing activities in lung cancer	118
**HEK 293 T cells, human lung spheroid cells**	EVs	Spike, GFP, RFP	Electroporation	Lung	Unique room-temperature-stable nanoparticle drug-delivery system, with enhanced bioavailability	119
**Bone-marrow-derived leucocytes**	EVs	GFP	Stability of Arc EVs via the A5U motif	Neurons	Enhances mRNA loading in the EV and target brain nerves *in vivo*, even in areas of low-grade chronic inflammation	120
**BL21 (DE3) Escherichia coli**	Outer membrane vesicles	EGFP, OVA or ADPGK	Active loading based on ClyA-L7Ae fusion protein	Dendritic cells	Repress melanoma growth and induce a 37.5% complete regression in colon cancer model	121
